# QUALITY OF LONG-TERM CARE FACILITIES AND COVID-19 OUTCOMES: A SYSTEMATIC REVIEW

**DOI:** 10.1093/geroni/igad104.3545

**Published:** 2023-12-21

**Authors:** Xiaochuan Wang, Gabriella Eyler

**Affiliations:** University of Central Florida, Orlando, Florida, United States; University of Central Florida, Orlando, Florida, United States

## Abstract

U.S. long-term care facilities (LTCFs) have been disproportionally and negatively impacted by the COVID-19 pandemic, reporting high shares of COVID-19 cases and deaths. It is critical to understand whether and how quality of these facilities is related to COVID-19 outcomes, to inform policy and practice to better protect the vulnerable LTCF residents and prepare for future outbreaks. Yet, research on this topic has primarily focused on nursing homes which has yielded inconsistent findings, and less is known in the assisted living care setting. To help address this gap, we systematically reviewed existing literature describing quality indicators and examining their impacts on COVID-19 outcomes in LTCFs. Using PRISMA guidelines, we searched electronic databases (e.g., PubMed) for peer-reviewed articles published in English between 2020 and 2023. The search yielded 1,487 unduplicated publications. After screening articles for relevance based on titles and abstracts, 123 were reviewed in full text. A total of 25 articles were included in this review. The included articles examined a range of quality indicators, such as CMS overall and sub-domain 5-star ratings and previous infection prevention and control deficiencies. Overall, the studies revealed mixed results regarding the association between quality and COVID-19 outcomes in LTCFs. Most studies found that higher quality is generally indicative of better COVID-19 outcomes, and some further suggested that the association varied across pandemic phases. Whereas another 7 studies found no significant relationship. This systematic review highlights the need for future research and evidenced-based policy and practice to better protect the at-risk LTCF resident population.

